# The Impact of Essential Oils Derived from Citrus Species to Control *Botrytis cinerea* and Their Potential Physiological Actions

**DOI:** 10.3390/plants14121859

**Published:** 2025-06-17

**Authors:** Sebastián Campos, Javier Espinoza, Juan Mauricio Fuentes, Ignacio Jofré-Fernández, Gonzalo Tortella, Diego Navarro, Andrés Quiroz, María Cristina Diez, Olga Rubilar, Paola Fincheira

**Affiliations:** 1 Environmental Nanobiotechnology Laboratory, Universidad de La Frontera, Av. Francisco Salazar 01145, Box 54-D, Temuco 4811230, Chile; s.campos05@ufromail.cl (S.C.); gonzalo.tortella@ufrontera.cl (G.T.); olga.rubilar@ufrontera.cl (O.R.); 2 Department of Chemical Sciences and Natural Resources, Faculty of Engineering and Sciences, Universidad de La Frontera, Av. Francisco Salazar 01145, Casilla 54-D, Temuco 4811230, Chile; andres.quiroz@ufrontera.cl; 3 Center of Excellence in Biotechnology Research Applied to the Environment (CIBAMA-UFRO), Faculty of Engineering and Sciences, Universidad de La Frontera, Av. Francisco Salazar 01145, Box 54-D, Temuco 4811230, Chile; cristina.diez@ufrontera.cl; 4 Doctoral Program in Science Mention Applied Cellular and Molecular Biology, Faculty of Agricultural Sciences and Environment, Universidad de La Frontera, Av. Francisco Salazar 01145, Box 54-D, Temuco 4811230, Chile; j.fuentes23@ufromail.cl; 5 Scientific and Technological Bioresource Nucleus (BIOREN-UFRO), Universidad de La Frontera, Av. Francisco Salazar 01145, Casilla 54-D, Temuco 4811230, Chile; ignacio.jofre@ufrontera.cl; 6 Department of Chemical Engineering, Faculty of Engineering and Sciences, Universidad de La Frontera, Av. Francisco Salazar 01145, Casilla 54-D, Temuco 4811230, Chile; 7 Tecnológico de Monterrey, Escuela de Ingeniería y Ciencias, Av. Gral. Ramón Corona No 2514, Colonia Nuevo México, Zapopan 45121, Mexico; diegonl@tec.mx; 8 Water Research Center for Agriculture and Mining—CRHIAM, ANID FONDAP Center, Victoria 1295, Concepción 4030000, Chile

**Keywords:** citrus essential oil, *Botrytis cinerea*, physiological mechanisms, solid lipid nanoparticles

## Abstract

*Botrytis cinerea* is one of the phytopathogenic fungi of the greatest economic importance worldwide. Essential oils (EOs) have been proposed as a sustainable alternative to reduce the growth of phytopathogenic fungi. Nevertheless, few studies exist about its mechanisms of action. This study evaluated the antifungal activity of EOs from *Citrus reticulata*, *Citrus limon*, *Citrus sinensis*, and *Citrus paradisi* peels and their encapsulation inside solid lipid nanoparticles (SLNs). Accordingly, *Citrus* EOs were mainly constituted by monoterpene hydrocarbons, where limonene was the most abundant in all EOs. *C. reticulata* and *C. limon* EOs reduced the mycelial growth at above 54% after 96 h. The other EOs did not significantly impact the phytopathogen. *C. reticulata* EO increased the hyphae damage by 40%, but the spore germination was reduced by only 8.34%. It also significantly increased the pH, the electrical conductivity, and the release of intracellular absorbing material and soluble proteins in *B. cinerea* cultures. Contrary, the esterase, mitochondrial, and succinate dehydrogenase activities decreased at above 50%. *C. reticulata* EO into SLN reduced the mycelial growth of *B. cinerea* by 90–97%. These results show that the EO of *C. reticulata* alters the physiological and metabolic activities of *B. cinerea* to reduce its growth.

## 1. Introduction

*Botrytis cinerea* is considered the second most important and destructive phytopathogen by the scientific community due to its economic and global impact on the loss of fruits and vegetables [1,2]. *B. cinerea* is a destructive necrotrophic plant pathogen that produces gray mold diseases, resulting in significant yield and quality losses (>50%) in vegetables and fruits [3]. This phytopathogen can infect a wide range of plant species, causing diseases in over 1400 plant species [2]. The germination of conidia and germ tubes on the surface of plants under appropriate environmental conditions facilitates infection in host tissue or cells [4]. The conidia of *B. cinerea* germinate on plant surfaces under appropriate temperatures (15–25 °C) and humidity (80%). The appressorium located at the tip of the germ tube invades host tissues or cells to complete the infection process. In the early stage, *B. cinerea* produces an asymptomatic biotrophic phase, which is followed by a necrotrophic phase as plant organs mature [5]. This fungus releases cell-wall-degrading enzymes, low-molecular-weight compounds, toxins, and phytotoxic metabolites that facilitate the infection process [6]. The control of gray mold is conventionally based on the application of synthetic fungicides, including hydroxyanilides, anilinopyrimidines, dicarboximides, and carboxamides, among others, which collectively account for approximately 10% of the global fungicide market [7,8]. Nevertheless, the adverse impact of synthetic fungicides on the ecosystem and human health has led to the search for sustainable alternatives [7,9]. In this context, essential oils (EOs) have emerged as an eco-friendly alternative to overcome the limitations and mitigate the harmful impact of synthetic fungicides in controlling fungal phytopathogens [10]. EOs are a natural mixture of hydrophobic secondary metabolites (around 20–60) with volatile and aromatic properties, including hydrocarbons, terpenes, and terpenoids produced naturally by plants or fruits [11]. EOs can be extracted from different plant organs, including flowers, seeds, leaves, fruits, roots, shoots, grasses, wood, and bark [12]. In this sense, EOs constitute an eco-friendly tool due to their biodegradable and biocompatible properties, which enable them to mitigate the adverse impacts of synthetic fungicides. EOs of citrus species are composed of secondary metabolites with an aromatic nature, comprising 85–99% volatile and 1–15% non-volatile components [13]. Volatile constituents contain monoterpene hydrocarbons (70–95%) and *D*-limonene. The antifungal mechanisms of EOs are associated with the alteration of ergosterol synthesis, modification of morphology, dysfunction of ATPase activity, and the production of reactive oxygen species [11]. Other studies have reported that the antifungal activity of EOs could be associated with the interaction and subsequent formation of hydrogen bonds between hydroxyl groups and the active site of fungal enzymes [14]. Recently, it has been reported that the EO of citrus species exhibits antimicrobial properties due to its capacity to damage cell membranes, inhibit the respiratory chain, and alter cell constituents (i.e., DNA, proteins, lipids) [13,15]. For example, Simas et al. [16] demonstrated that EOs of *Citrus limon* and *Citrus limonia* at 312 μg mL^−1^ completely inhibited the mycelial growth of *B. cinerea* due to the disorganization in the fungal cell membrane [16]. Besides, the vapor phase released from 625 µL L^−1^ of EO of lemongrass inhibited the growth of *B. cinerea* [17]. Nevertheless, studies on the impact of the EOs of citrus on controlling *B. cinerea* are scarce, and their action mechanisms have not been fully elucidated. Furthermore, the application of EO in agriculture is scarce due to its lipophilic nature and sensitivity to environmental conditions, which prevents its direct application. In this sense, solid lipid nanoparticles (SLNs) (size: 50 to 1000 nm) have been proposed for the encapsulation of EO due to it immobilizing and protecting the active agent within solid lipids [18]. The SLNs are characterized by their biodegradable nature, stability, water solubility, and protection of the active agent against chemical, photochemical, and oxidative degradation [19]. In this context, this study focused on (1) evaluating the EOs from the peel of *Citrus reticulata* Blanco (Mandarin), *Citrus limon* Risso (Lemon), *Citrus sinensis* L. (Sweet orange), and *Citrus paradisi* Macfad. (Grapefruit) for controlling the growth of *B. cinerea*, (2) elucidating their potential action mechanisms of EO of citrus species, and (3) prospecting SLN to apply EO in agricultural systems.

## 2. Results

### 2.1. Characterization of the Essential Oils

EOs from peels of *C. reticulata*, *C. limon*, *C. paradisi*, and *C. sinensis* were analyzed by gas–liquid chromatography coupled with mass spectrometry (GC/MS) (Table 1). Eleven, twelve, nine, and eight compounds, corresponding to 99.64%, 99.29%, 99.95%, and 99.92% of the detected compounds were identified in the *C. reticulata*, *C. limon*, *C. paradisi*, and *C. sinensis* EOs, respectively.

Six monoterpene hydrocarbons (99.36%) and five sesquiterpenes (0.28%)—four non-oxygenated (0.21%) and one oxygenated (0.07%)—were present in the *C. reticulata* EO. Similarly, seven monoterpene hydrocarbons (97.78%) and five sesquiterpenes (1.51%)—four non-oxygenated (1.42%) and one oxygenated (0.09%)—were in the *C. limon* EO. Four monoterpene hydrocarbons (99.55%) and five sesquiterpenes (0.40%)—four non-oxygenated (0.38%) and one oxygenated (0.02%)—were in the *C. paradisi* EO. Six monoterpenes (99.88%)—four non-oxygenated (95.97%) and two oxygenated (3.91%)— and two sesquiterpene hydrocarbons (0.04%) were present in the *C. sinensis* EO.

The monoterpene hydrocarbon limonene was the most abundant compound in all *Citrus* EOs, followed by *γ*-terpinene (26.29%) in *C. reticulata* EO, and by *β*-pinene (21.89%) and *γ*-terpinene (12.85%) in *C. limon* EO. The major content of limonene was found in C. *paradisi* and *C. sinensis* EOs, with 98.51% and 95.23%, respectively (Table 1).

### 2.2. Inhibition of Mycelial Growth and Spore Germination

**Figure 1:**
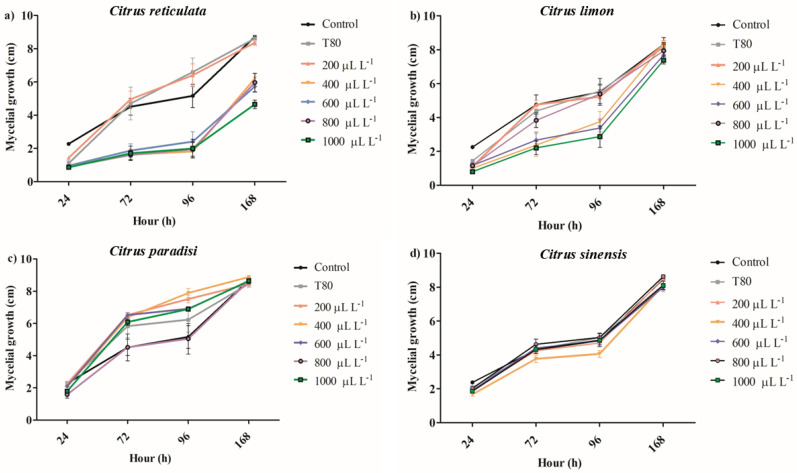
The impact of different concentrations of essential oils of (**a**) *Citrus reticulata*, (**b**) *Citrus limon*, (**c**) *Citrus paradisi*, and (**d**) *Citrus sinesis* on mycelial growth of *Botrytis cinerea*. (mean values ± standard error n = 4).

**Figure 2:**
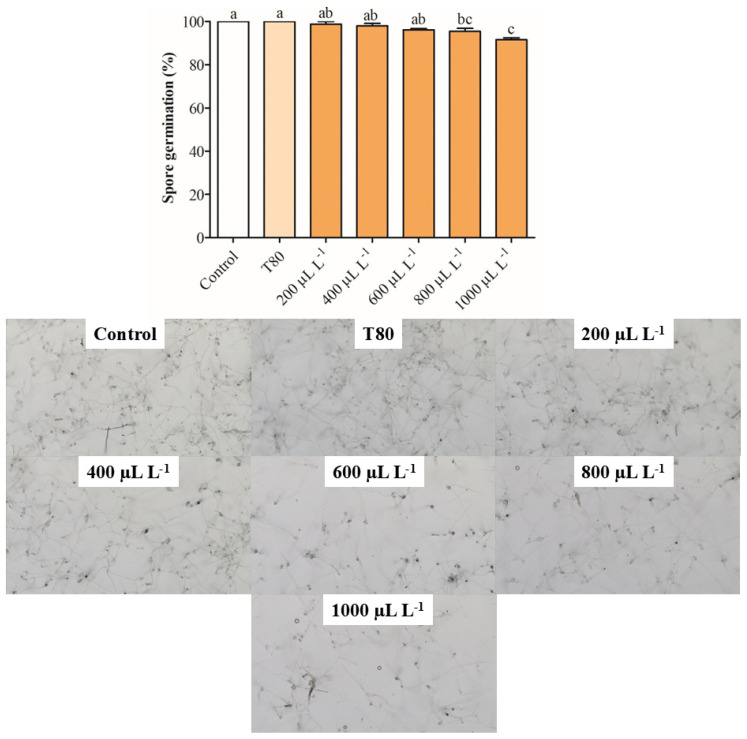
Inhibition of spore germination of *B. cinerea* exposed to different concentrations of *C. reticulata* EO. Different letters above bars indicate significant differences according to the Tukey test (*p* < 0.05) (mean values ± standard error, n = 3).

The EO of *C. reticulata* at 1000 μL L^−1^ reduced the mycelial growth between 62 and 64% after 72 and 96 h of exposure (Figure 1a). Concentrations of 400, 600, and 800 μL L^−1^ of the same EO similarly reduced the mycelial growth of *B. cinerea* on hours 72 and 96 and reduced the antifungal activity on hour 168. The EO of *C. limon* at concentrations from 800 to 1000 μL L^−1^ inhibited the mycelial growth of *B. cinerea* between 44 and 54% on hour 72, but the antifungal activity decreased on hour 168 (Figure 1b). The lower concentrations of EO of *C. limon* (400 and 600 μL L^−1^) had a similar antifungal activity behavior. It was observed that the EO of *C. paradisi* at 400 μL L^−1^ increased the mycelial growth between 41 and 56% on hours 72 and 96, respectively (Figure 1c). Similarly, the other concentrations produced an increase in the mycelial growth of *B. cinerea*. Finally, the application of EO of *C. sinensis* did not affect mycelial growth during the 168 h evaluated (Figure 1d). In summary, the results showed that the EO of *C. reticulata* presented the best activity in reducing mycelial growth, whereby it was selected for the following assay. Nevertheless, the spore germination was slightly reduced by around 8.34% with exposure for 24 h at 1000 μL L^−1^ of EO of *C. reticulata* (Figure 2).

### 2.3. Dry Weight, Electrical Conductivity, and pH

**Figure 3:**
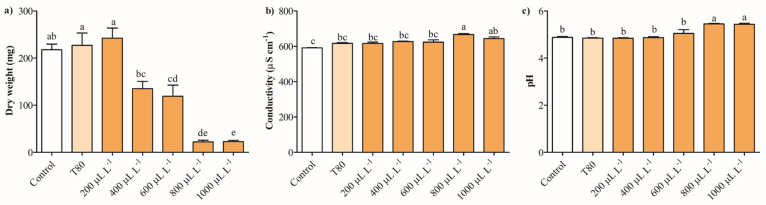
Effects of *C. reticulata* EO on (**a**) dry weight and (**b**,**c**) release of cell constituents of *B. cinerea* measurement through extracellular pH and conductivity. Different letters above bars indicate significant differences according to the Tukey test (*p* < 0.05) (mean values ± standard error, n = 3).

Figure 3a shows that the dry weight of *B. cinerea* decreased by approximately 89% with exposure to 800 and 1000 μL L^−1^, while concentrations of 400 and 600 μL L^−1^ reduced this parameter by 37–45%. The fungus exposure at 200 μL L^−1^ of EO of *C. reticulata* had no antifungal effect on *B. cinerea*. The electrical conductivity and pH of *B. cinerea* cultures were measured to investigate potential alterations in the cell membrane with exposure to the EO. The electrical conductivity in cultures of *B. cinerea* slightly increased by 5–12% with 800 and 1000 μL L^−1^ of EO of *C. reticulata*, respectively (Figure 3b). Meanwhile, concentrations from 200 to 600 μL L^−1^ did not change significantly the electrical conductivity of the cultures of *B. cinerea*. Regarding pH, the cultures of *B. cinerea* exposed to 800 and 1000 μL L^−1^ of EO increased this parameter by 11%, while concentrations between 200 and 600 μL L^−1^ had no impact on *B. cinerea* cultures (Figure 3c).

### 2.4. The Release of Intracellular Constituents and Potential Physiological Mechanisms

**Figure 4:**
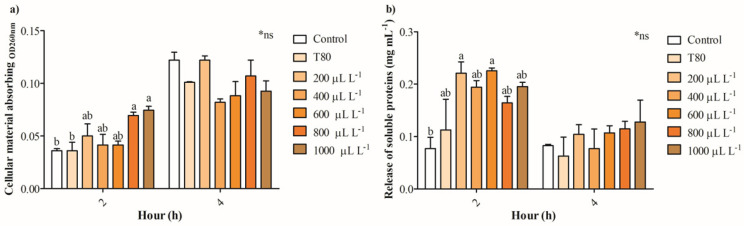
The impact of essential oil from *C. reticulata* peels on (**a**) intracellular absorbing material OD_260nm_ and (**b**) extracellular soluble proteins. Different letters above bars indicate significant differences according to the Tukey test (*p* < 0.05) (mean values ± standard error, n = 3). *ns indicate no statistically significant differences.

**Figure 5:**
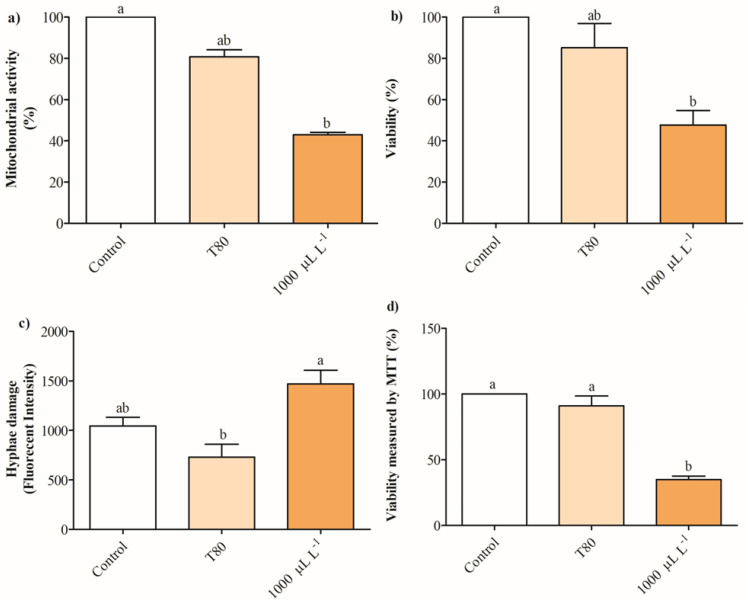
The potential action mechanisms of the *C. reticulata* EO to suppress the growth of *B. cinerea* were measured through (**a**) mitochondrial activity, (**b**) viability, (**c**) hyphae damage, and (**d**) intracellular esterases measured by the MTT technique. Different letters above bars indicate significant differences according to the Tukey test (*p* < 0.05) (mean values ± standard error, n = 3).

Figure 4a shows that the release of intracellular absorbing material OD_260nm_ in cultures of *B. cinerea* exposed to 200- 600 μL L^−1^ of EO of *C. reticulata* partially increases between 13 and 38%, while concentrations of 800 and 1000 μL L^−1^ enhanced 1-fold after 2 h. No significant differences were found in the release of intracellular absorbing material OD_260nm_ of cultures of *B. cinerea* after 4 h of exposure to EO of *C. reticulata.* Similarly, the release of intracellular soluble proteins increased significantly in cells of *B. cinerea* exposed to 200 and 600 μL L^−1^ of EO of *C. reticulata* with 2 h of exposure, and no differences were found after 4 h (Figure 4b). Additionally, Mitotracker orange, Propidium iodide, and Calcein AM fluorescence staining were performed to explore mitochondrial activity, cell viability (hyphae damage), and esterase activity of cultures of *B. cinerea* exposed to EO of *C. reticulata*. Figure 5 shows that the cultures of *B. cinerea* exposed to EO of *C. reticulata* at 1000 μL L^−1^ decreased mitochondrial activity, esterase activity, and succinate dehydrogenase by 57%, 52%, and 65%, respectively. Simultaneously, the hypha damage of *B. cinerea* increased by 40% with the application of EO.

### 2.5. Characterization and Evaluation of SLN Loaded with the EO of C. reticulata

**Figure 6:**
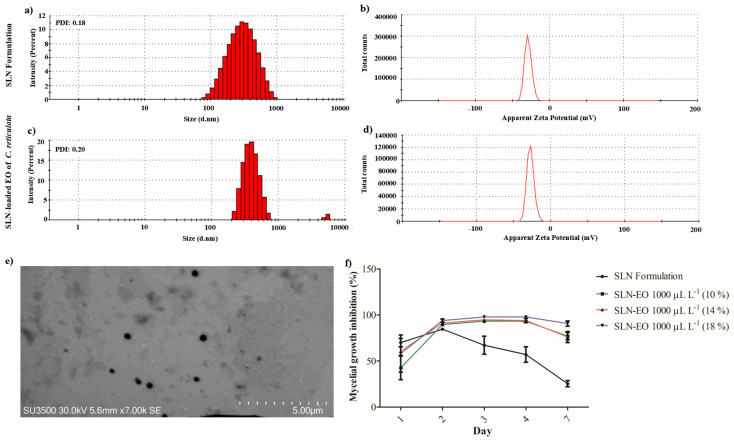
Physicochemical characterization and antifungal activity of solid lipid nanoparticles (SLNs) loaded with *C. reticulata* EO. (**a**,**c**) Hydrodynamic size distribution and (**b**,**d**) ζ-potential of SLNs, (**e**) representative photograph of SLN captured by scanning transmission electron microscopy (STEM), and (**f**) the mycelial growth inhibition of *B. cinerea* modulated the controlled release of the EO of *C. reticulata* from SLN (mean values ± standard error, n = 3).

SLN formulation and *C. reticulata* EO-loaded SLN had a mean hydrodynamic size of 262 and 406 nm, respectively. Both SLNs demonstrated high homogeneity in particle size, as indicated by their polydispersity index (PDI) values, which ranged from 0.18 to 0.20 (Figure 6a,c). Moreover, the encapsulation efficiency ranged from 90 to 93%. Meanwhile, the δ-potential of SLNs obtained had values from −27.1 to −29.0 mV, showing similar stability and supporting that the encapsulation of EO of *C. reticulata* does not modify the stability of the particle (Figure 6b,d). Further, the stability of the SLN formulation was also measured for 3 weeks, with values ranging from −17.7 mV to −25.5 mV, indicating that hydrophobic compounds could be encapsulated stably over time.

The photographic capture by scanning transmission electron microscopy (STEM) evidenced the spherical shape and homogeneous surface of the SLN formulation (Figure 6e). Figure 6e shows that on day 2, there was a small significant effect of *C. reticulata*-loaded SLN compared to the formulation of SLN (94% versus 84%). It was evidenced that SLN-loaded with EO of *C. reticulata* reduced the mycelial growth of *B. cinerea* between 90 and 97%. In contrast, the impact of the SLN formulation decreased significantly over time, from 67% to 25% (Figure 6f).

## 3. Discussion

Fungi species are responsible for 70–80% of plant diseases caused by phytopathogenic microorganisms, which reduce the growth and yield of agricultural crops [20,21]. Phytopathogenic fungi release low-molecular-weight secondary metabolites, which produce disease symptoms in vegetable tissues, including chlorosis, necrosis, growth inhibition, and leaf spotting [22]. In particular, *B*. *cinerea* is considered the second most important phytopathogenic fungus worldwide due to its widespread distribution, resulting in significant losses during the pre- and post-harvest stages of vegetables and fruits of economic importance [1,6]. Until now, synthetic fungicides have demonstrated the greatest efficiency in controlling phytopathogenic fungi, but their adverse effects on the ecosystem and human health limit their application [21]. Studies on formulating products based on natural molecules have recently increased. In this context, the application of EO has emerged as a sustainable alternative to reduce the infection of *B. cinerea* and the application of synthetic fungicides due to it is generally recognized as safe (GRAS) [23]. Studies have shown the efficient antifungal activity of EO extracted from plants such as thyme, oregano, clove, lavender, rosemary, and eucalyptus [24,25]. However, studies comparing the efficiency of EO of citrus species in inhibiting the growth of *B. cinerea* are scarce even though they have been reported as an important by-product of citrus processing due to their several biological activities, including broad-spectrum antimicrobial, anti-viral, anti-insecticidal, anti-inflammatory, anti-cancer, and immunomodulatory, among others [26]. The effects of EOs from citrus species have been reported in fungal species, including *Penicillium italicum*, *Penicillium expansum*, *B. cinerea*, *Geotrichum citri-aurantii*, and *Pyricularia oryzae*, among others [27,28]. Based on the above, this study was focused on evaluating EOs from the peel of citrus species, prospecting the potential physiological mechanisms and nanoencapsulation techniques to apply in agricultural systems.

The genus *Citrus*, belonging to the Rutaceae family, consists of various species commonly known as mandarins, oranges, lemons, and pomelos. These species are categorized differently under different classification systems. Swingle’s classification identified 16 distinct species, while Tanaka’s horticultural classification recognized a more extensive 162 species [29,30]. Despite its complicated phylogeny situation, citrus peel is a by-product with valuable applications due to its rich chemical composition. The potential of citrus peel lies mainly in EOs, which are characterized by their strong and pleasant aroma and are used in various foods, beverages, and pharmaceuticals [31]. The chemical composition of *Citrus* EOs has been extensively studied for the last decades [32]. These EOs are complex mixtures of volatile organic compounds rich in monoterpene and sesquiterpene hydrocarbons [33]. Particularly, the EOs obtained from citrus peel always show limonene, a monoterpene hydrocarbon, as the most abundant compound, generally representing about 60–95% of the oil [32]. The EOs analyzed in the present study were no exception. Here, limonene was also the most abundant compound in all analyzed *Citrus* EOs, representing 66.6%, 59.7%, 98.5%, and 95.2% of the *C. reticulata* (Mandarin), *C. limon* (Lemon), *C. paradisi* (Grapefruit), and *C. sinensis* (Sweet orange) EOs, respectively. The latter two oils showed the major content of limonene. Limonene was followed by *γ*-terpinene (26.3%) in *C. reticulata* EO, and by *β*-pinene (21.9%) and *γ*-terpinene (12.9%) in *C. limon* EO. It is in accordance with literature where limonene is the most common compound, followed by *β*-myrcene, 3-carene, *α*-pinene and *β*-pinene, *γ*-terpinene, linalool, *β*-terpineol, and *β*-citronellol [32].

Comparing among species, Dosoky and Setzer [26] reported that the peel of mandarin fruits contains mainly monoterpenes hydrocarbons, accounting for 86.62% of the oil, and lower than levels detected in other *Citrus* fruits like sweet orange (83.9–95.9%), bitter orange (89.7–94.7%), and grapefruit (84.8–95.4%). In the present study, *C. reticulata* EO contained 99.4% of monoterpene hydrocarbons, like other *Citrus* fruits. Moreover, monoterpenes hydrocarbons in the peel of lemon, grapefruit, and sweet orange represented 96.0–99.5% of the oils, according to the literature [26].

Particularly to *C. reticulata* (Mandarin) EO studied here, limonene was the most abundant compound (66.6%), followed by *γ*-terpinene (26.3%), *α*-pinene (2.5%), *β*-pinene, (2.2%), *o*-cymene (1.1%), and *β*-myrcene (0.6%). This composition is quite similar to the reported by Dosoky and Setzer [26], where the most prevalent terpene in mandarin was limonene (95%), followed by γ-terpinene (16.4–22.7%), *α*-pinene (2.0–2.7%), *β*-pinene, (1.4–2.1%), *β*-myrcene (1.5–1.8%), and linalool (0.67%). However, the content of any component of EO in mandarin and other fruits may differ depending on the variety, ripening stage, extraction method, and plant structure [34].

In relation to biological activities, the EOs of citrus species studied here had a differential activity to inhibit the mycelial growth of *B. cinerea*. For example, the EO of *C. sinensis* did not affect mycelial growth, while the EO of *C. paradisi* stimulated the mycelial growth at concentrations greater than 200 µL L^−1^. The EO of *C. limon* and *C. reticulata* reduced the mycelial growth after 72 and 96 h of exposure. It notes that only EO of *C. reticulata* at 1000 µL L^−1^ maintained the reduction of mycelial growth of *B. cinerea* during the period of evaluation. These results are in concordance with the reported by De-Montijo-Prieto et al. [35], where the EO of *C. sinensis*, *C. limon*, and *C. paradisi* had a low capacity to inhibit mycelial growth (3.4–18.4%). However, this study differs in its antifungal activity, reporting a 3.6% reduction in the mycelial growth of *B. cinerea*. Additionally, the results are different from those reported by Badawy et al. [36], who evidenced that EO of *C. limon* and *C. sinensis* (313–431 mg L^−1^) had the best antifungal activity against *B. cinerea* compared to *C. paradisi* (809 mg L^−1^) according to effective concentration using 50% growth inhibition (EC_50_) values. Other studies have shown that concentrations of 11.3% *v*/*v* × 10^−2^ of EO of *C. sinensis* and *C. limon* inhibited mycelial growth between 88% and 99% after 3 days of exposure [37]. Furthermore, higher concentrations of EO of *C. limon* at 20% and 35% reduced mycelial growth by 100% after 7 days of incubation [38]. The volatile exposure to 1.25 µL of EO of *C. limon* for plates reduced the mycelial growth of *B. cinerea* by around 70% (minimum inhibitory concentration: 312 µg mL^−1^) [16]. In summary, the results of mycelial growth inhibition in *B. cinerea* show a differential behavior compared to other studies, which can be attributed to exposure contact, EO concentration, and the evaluation period. It is noteworthy that the EO of *C. reticulata* was selected for the following assays because it showed the greatest activity in inhibiting the mycelial growth of *B. cinerea*. Unfortunately, the selected EO had a low effect on reducing spore germination of *B. cinerea*, which is in concordance with the finding reported by Badawi et al. [36].

The studies have indicated that inhibiting mycelial growth and spore germination strongly depends on the EO and its chemical composition. Recently, Lin et al. [39] reported that limonene can act as an antifungal agent reducing the growth of many phytopathogen fungi through the damage of cell walls and cell membranes, promoting the production of ROS, altering energy metabolism and respiration, producing DNA damage and the leakage of intracellular contents. However, in the present study, the EOs of *C. paradisi* and *C. sinensis*, which presented the highest amount of limonene, were not capable of inhibiting the mycelial growth of *B. cinerea*. Contrary, EOs from *C. reticulate* and *C. limon* inhibited mycelial growth. These EOs were constituted by lesser amounts of limonene but also presented γ-terpinene as well as other minor compounds. Additionally, γ-terpinene was absent in *C. paradisi* and *C. sinensis* EOs. γ-terpinene was found in the EOs from pharmacologically active plants like *Citrus deliciosa* Tenore, *Origanumonites* L., and *Protiumicicariba* (DC.) Marchand. Due to the liposolubility of γ-terpinene, it can be easily absorbed through biological members [40]. Then, the above would suggest that a combination of γ-terpinene and limonene could be responsible for the inhibition of the mycelial growth of *B. cinerea*. It is known that the effects of single compounds are not necessarily the same as the whole mixture, the interactions between molecules may lead to antagonistic, additive/non-interactive, or synergistic effects [41]. In this case, limonene mixed with γ-terpinene and other minor compounds in the EO showed a synergistic effect. Moreover, minor compounds also could be involved in antifungal activity. *α*-Pinene and *β*-pinene, both constituents of the *C. reticulate* and *C. limon* EOs, have shown antifungal activity against other phytopathogens. Lee et al. [42] showed that *α*-pinene reduced the growth of *Colletotrichum gloeosporioides* by 100% with exposure to doses of 10, 5, 2.5, and 1.25 µL by increasing the overproduction of ROS, the disruption of the cell membrane, and decreasing the ergosterol content. Furthermore, *β*-pinene has been reported for its efficient antifungal activity against *Rhizoctonia solani* (IC_50_ values of 2.439 and 1.857 µg mL^−1^) [43]. However, further studies evaluating the antifungal activity of pure terpenes against *B. cinerea*, considering mixtures of those at different concentrations could be really valuable to elucidate the antifungal mechanisms.

Notwithstanding the above, the presence of monoterpenes in EO has an important role in inhibiting mycelial growth because it increases the lipidic peroxides, including alkoxyl, hydroxyl, and alkoperoxyl radicals [36]. In addition, phenolic compounds (i.e., thymol, eugenol, and carvacrol) contribute to the alteration of function and permeability of cell membrane proteins [44]. Phenols with free -OH group can modify amino acid residues of proteins and interact with protein targets of fungal cells [45]. Terpenes and phenolic compounds enable EO to accumulate in the hydrocarbon molecules of the lipid bilayer of fungal cells, facilitating its entry into fungal cells [10]. The significant reduction in the dry weight of *B. cinerea* with the exposure to 800 and 1000 µL L^−1^ of EO of *C. reticulata* for 7 days supported efficient antifungal activity. Once the EO crosses the cell membrane, it can alter various physiological and metabolic functions, thereby reducing the growth of the phytopathogenic fungi. In this sense, this study determined the electrical conductivity and pH of cultures of *B. cinerea* exposed to different concentrations of EO of *C. reticulata* as indirect parameters to analyze cell membrane permeability. The fungal cell membrane maintains cellular and molecular functions to ensure adequate homeostasis and protection for survival [45,46]. Therefore, alterations in the fluidity and integrity of the fungi cell membrane respond to the initial adverse impact of *C. reticulata* EO to trigger the alteration in intracellular compartments.

The extracellular pH and conductivity evidenced that treatments of 800 and 1000 µL L^−1^ of EO of *C. reticulata* produce an alteration in the membrane cell of *B. cinerea*, which is attributed to an intracellular leakage of Mg^2+^, K^+^, and Ca^2+^ [47]. Pavoni et al. [48] reported that an increase in extracellular pH in fungi cultures is due to an enhancement of ionic permeability and alteration of osmotic pressure. Otherwise, the increase in electrical conductivity can be attributed to alterations in the cell membrane and the release of intracellular content [49,50]. The increase of these parameters is in concordance with the results obtained with the exposure of *B. cinerea* cultures to EO of *Litsea cubeba*, *O. vulgare*, and *T. vulgaris* [51,52]. Additionally, the cell membrane integrity of *B. cinerea* was evaluated by measuring the release of cellular material, as indicated by an OD_260nm_ absorption and soluble proteins [50]. The release of intracellular material or the leakage of cytoplasmic content is attributed to an imbalance in intracellular osmotic pressure resulting from alterations in cell membrane permeability [24]. The exposure of *B. cinerea* cultures to high concentration of EO of *C. reticulata* increased the release of intracellular constituents, confirming the disruption of cell membrane integrity after 2 h. Both parameters are indicators of severe damage in fungi cell membranes, supporting the efficient antifungal activity of EO of *C. reticulata*. These results are attributed to an imbalance in osmotic pressure between the extracellular and intracellular compartments due to the loss of permeability, which was evidenced by increased pH and electrical conductivity in cultures of *B. cinerea* [53]. The results showed that the EO of *C. reticulata* 1000 µL L^−1^ has the best antifungal activity against *B. cinerea* through the alteration of the cell membrane permeability and the release of cytoplasmic content. Therefore, this concentration was used to investigate the potential mechanisms of action triggered by the EO of *C. reticulata* in inhibiting *B*. *cinerea*.

Propidium iodide was applied to determine the hyphae damage of *B. cinerea* exposed to EO, where the dye binds to double-stranded DNA in dead cells due to the alteration in the cell membrane [54]. Cell membrane damage in *B. cinerea* increased by 40% with the exposure to EO of *C. reticulata*, confirming the alteration of cell membrane integrity. Similar results were obtained with the exposure of *B. cinerea* cells to vapor release of EO of *Cymbopogon citratus*, *T. vulgaris*, and *O. heracleoticum* at 50 µL L^−1^ [55]. In addition, the EO of *Solidago canadensis* L. at 16.5 mL L^−1^ produced the same impact on the cell membrane integrity of *B. cinerea* [56]. Additionally, Calcein AM was applied to *B. cinerea* cultures exposed to EO of *C. reticulata* to determine cell viability through esterase activity. Calcein AM is a cell-penetrating fluorescent dye used to determine cell viability in eukaryotic cells. Non-fluorescent calcein AM is converted to green fluorescent calcein in living cells, which is followed by the hydrolysis of the acetoxymethyl ester by intracellular esterases [57]. The results showed a decrease in intracellular esterase activity of ~ 50% in cultures of *B. cinerea* exposed to the EO. This result is in concordance with the exposure of *B. cinerea* to EO of *T. vulgaris* and *O. vulgare* in the range concentration from 300 to 500 µL L^−1^ [52].

Previous studies have demonstrated that EO leads to abnormal metabolisms due to the dysfunction of mitochondrial activity [45]. The results obtained in this study evidenced that 1000 µL L^−1^ of EO of *C. reticulata* decreased 57% mitochondrial activity, suggesting a disruption in the respiratory chain and tricarboxylic acid (TCA) cycle pathways. The EO can alter the ATPase and dehydrogenase activities, decreasing the energy production and biochemical reactions in mitochondria [58]. It has been reported that phenolic compounds in the EO cause hyperpolarization and dysfunction of mitochondria, leading to the depletion of adenosine triphosphate (ATP) [59]. This review also indicated that EO can modulate mitochondrial activity by altering mitochondrial REDOX balance, inhibiting mitochondrial enzymes, disrupting mitochondrial membranes, and suppressing oxidative phosphorylation. Previously, Li et al. [60] reported that the EO of tea tree increased the mitochondrial membrane permeability and decreased enzymatic activities of the TCA cycle, including isocitrate dehydrogenase, d-ketoglutarate dehydrogenase, citrate synthetase, malic dehydrogenase, ATPase, and succinate dehydrogenase. In this sense, succinate dehydrogenase activity in *B. cinerea* exposed to the EO was determined using the 3-(4,5-Dimethylthiazol-2-yl) 2,5-diphenyltetrazolium (MTT) technique. MTT reagent can cross through the cell and mitochondrial inner membranes of viable cells and be reduced to formazan in living cells [61]. The EO of *C. reticulata* decreased the succinate dehydrogenase activity of *B. cinerea* by 65%, confirming an alteration in mitochondrial activity due to the alteration of essential enzymes to maintain the cell metabolic activity. Succinate dehydrogenase, also known as complex II, participates in the TCA cycle and electron transport chain, which is also a target enzyme of some chemical fungicides [62]. Specifically, the TCA cycle corresponds to metabolic reactions in which mitochondria generate ATP in organisms that are oxidative [63]. Similarly, the EO of tea tree between 1 and 2 mL L^−1^ decreased the succinate dehydrogenase activity in *B. cinerea* [60,64]). Additionally, Hu et al. [58] demonstrated that the EO of *Curcuma longa* inhibited succinate dehydrogenase and other dehydrogenase activities. In summary, the results obtained in this study evidenced that the EO of *C. reticulata* had a significant antifungal activity to reduce the growth of *B. cinerea* by altering physiological functions, including the permeability of cell membrane, the release of intracellular material, the dysfunction in esterase activity, and altering the mitochondrial activity and enzymes related to the TCA cycle.

Once the antifungal action of EO of *C. reticulata* was proven, its application was prospected through its encapsulation in lipid nanoparticles. The controlled release of EO of *C. limon* from nanosystems exhibited greater fungicidal potential than that of EO alone [65]. Furthermore, vapor-based citrus EOs offer greater advantages than direct application by reducing toxicity and facilitating application [66]. In this study, we used SLN for the encapsulation of EO of *C. reticulata* due to its ability to protect and control the release of compounds, according to its interaction with the lipid matrix [18]. Additionally, SLN is characterized by its biodegradability, low production cost, and approval by the Food and Drug Administration (FDA) for its use in biological systems. In this study, SLNs were formulated using Tween 80 and glyceryl tristearate with a high-revolution homogenization and ultrasonication methodology. The incorporation of 1000 µL L^−1^ of EO of *C. reticulata* into SLN increased the hydrodynamic size from 263 to 406 nm. On the contrary, Fuentes et al. [67] showed that the encapsulation of EO of *Mentha piperita* at 500, 700, and 900 µL L^−1^ reduced the hydrodynamic size. It has been reported that the size of SLN depends on the interaction of the compounds with the lipid matrix [68]. The homogeneity of SLN size was measured by PDI, where values less than 0.1 showed monodispersion, PDI values from 0.1 to 0.4 represent moderate polydispersity, and values higher than 0.4 are highly polydisperse [69]. The SLN samples exhibited a PDI between 0.18 and 0.20, indicating good particle size homogeneity and minimal aggregation in the colloidal solution [70]. Meanwhile, high values of ζ-potential (−27.1 to −29.0 mV) obtained in this study reflected the stability of EO of *C. reticulata*-loaded SLN due to the high energy barrier and favored good stability.

The antifungal activity against *B. cinerea* was evaluated once the characterization and stability of SLN were verified. In recent years, studies have demonstrated the efficient antifungal activity of EO released from lipid nanoparticles [71]. In this study, the results supported the controlled release of EO of *C. reticulata* from SLN exhibited stable antifungal activity during the evaluation period. This result concurs with previous reports from other studies. For example, the EO of cuminum and cyminum incorporated into nanoemulsions had significant antifungal activity against *Aspergillus flavus* [72]. Furthermore, clove, thyme, cinnamon, and rosemary EO-loaded nano-structured lipid carriers decreased the mycelial growth of *Fusarium oxysporum* [73]. Similarly, Vakili-Ghartavol et al. [74] showed that the EO of *Mentha × piperita* L. encapsulated into SLN had a significant antifungal activity against *Rhizoctonia solani* and *Rhizopus stolonifer*.

In summary, this study provided important evidence about the significant activity of the EO of *C. reticulata* to reduce the mycelial growth of *B. cinerea* compared to the EO of *C. sinensis*, *C. paradisi*, and *C. limon*. The EO of *C. reticulata* altered the physiological and metabolic activities of *B. cinerea*, modifying the biological process to reduce mycelial growth. Additionally, the results evidenced that the encapsulation of EO of *C. reticulata* into SLN can be an eco-friendly alternative to prospect its application in agricultural systems to mitigate the use of synthetic fungicides. However, further studies considering pure compounds and mixtures at different concentrations could be valuable to elucidate the antifungal mechanisms of the EOs against *B. cinerea*.

## 4. Materials and Methods

### 4.1. Characterization of the Essential Oils by GC/MS

The EOs from peels of *C. reticulata*, *C. limon*, *C. paradisi*, and *C. sinensis* were acquired commercially from Qenkón Aromatherapy^®^ company (Santiago, Chile) (https://qenkon.cl/, accessed on 14 June 2025). The analysis of constituents from these EOs was conducted by gas chromatography/mass spectrometry (GC/MS) using a Thermo Scientific TRACE 1300 Series gas–liquid chromatography (Waltham, MA, USA) coupled to a Thermo Scientific ISQ 7000 single quadrupole mass detector, with an integrated data system (Xcalibur 4.2.47, Thermo Fisher Scientific Inc., Waltham, MA, USA). A 30 m long BPX5 capillary column (0.25 μm film thickness × 0.25 mm inner diameter, SGE Forte, Trajan Scientific and Medical, Ringwood, Victoria, Australia) was used for separation, and He at 1.00 mL min^−1^ was the carrier gas. The operating conditions included the following: Injector, transfer line, ion source, and detector temperature: 250 °C; oven temperature program: Hold at 40 °C for 1 min. After, it increased to 250 °C at 5 °C min^−1^, and then maintained for 5 min. The mass spectra were obtained at an ionization voltage of 70 eV. The recording conditions employed a scan time of 1.5 s and a mass range of 30 to 400 amu. The identification of the compounds was carried out by comparison of the mass spectra for each detected compound with those in the NIST ver. 2.0 library database (NIST, Gaithersburg, MD, USA). In others, by comparison of the retention times and the mass spectra for each detected compound with those of standards [75], and by comparison of the calculated retention index with those reported in the literature [76] considering the same type of stationary phase. The retention indices were calculated by means of C9–26 n-alkane standards (100 µg mL^−1^ in *n*-hexane) (Sigma-Aldrich, St. Louis, MO, USA) using the equation described by Kovats and Keulemans [77].

### 4.2. B. cinerea Strain

*B. cinerea* (CCCT 21.01) was acquired from the Chilean Culture Collection of Type Strains (CCCT-UFRO) belonging to Scientific and Technological Bioresource Nucleus BIOREN at Universidad de La Frontera (Temuco, Chile) and maintained on potato dextrose agar medium (PDA) (Difco^®^ Laboratories, Detroit, MI, USA) at 24 °C ± 2 °C under dark conditions.

### 4.3. Inhibition of B. cinerea Mycelium Growth

The inhibition of *B. cinerea* mycelial growth was performed by the Kirby–Bauer methodology. The inoculum of *B. cinerea* was previously grown on PDA (Difco^®^ Laboratories, Detroit, MI, USA) for 7 days at 25 °C in dark conditions. Then, a fungal disc (5 mm) of mycelium inoculum was extracted from the edge of the colony culture and placed in the center of a Petri dish (Greiner Bio-One, Kremsmünster, Austria) containing PDA amended with treatments. The treatments were solutions of the *C. limon*, *C. paradisi*, *C. sinensis*, and *C. reticulata* EOs (Qenkón Aromatherapy company, Santiago, Chile) at 200, 400, 600, 800, and 1000 µL L^−1^ in 0.05% Tween-80 (Difco^®^ Laboratories, Detroit, MI, USA). Petri dishes with PDA containing 0.05% Tween-80 inoculated with a 5 mm disc of fungal mycelium were considered the first control, and inoculated Petri dishes not exposed to EO or 0.05% Tween-80 were the second control. After, the Petri dishes were sealed with Parafilm (Heathrow Scientific^®^, Vernon Hills, IL, USA) and placed in an incubator (Labwit, Shanghai, China) at 25 °C ± 2 °C for 7 days in dark conditions [67]. Five replicates were considered for each treatment and control.

### 4.4. Inhibition of Spore Germination of B. cinerea

The inoculum of *B. cinerea* was previously grown on PDA for 14 days for conidia production. Sterile distilled water (3 mL) was added to the Petri dish, and the fungi culture was gently scraped to obtain the spores. A solution of spores was adjusted to 10^4^–10^5^ conidia mL^−1^ on a hemocytometer slide under an optical microscope Leica DM5000 integrated with an ICC50 camera (Leica Microsystems, Wetzlar, Germany). Then, 40 µL of spore solution at 10^4^–10^5^ conidia mL^−1^ was placed on the surface of microscopic slides containing 1 mL of PDA with the selected EO solution at 200, 400, 600, 800, and 1000 µL L^−1^ in 0.05% Tween-80. The first and second controls described in point 4.3 were also used here. Three slides for each treatment were used and placed in Petri dishes. Next, Petri dishes were sealed with Parafilm^®^ and incubated at 25 °C ± 2 °C under dark conditions. The spore germination was evaluated after 24 h using a Leica DM5000 optical microscope integrated with an ICC50 camera (Leica Microsystems, Wetzlar, Germany). Five focus areas were analyzed for each replicate. The fungal conidia were considered germinated when the germ tube lengths were equal or when multiple germ tubes were derived from the same conidia [78]. Three replicates were implemented for each treatment and control.

### 4.5. Evaluation of Dry Weight, pH, and Electrical Conductivity to Investigate Potential Alterations in the Cell Membrane

Dry weight, pH, and electrical conductivity were measured to prospect the physiological impact of selected EO on the culture of *B. cinerea*. For this, 100 μL of conidial solution (10^4^–10^5^ conidia mL^−1^) was added to 50 mL of potato dextrose broth (PDB) (Difco^®^ Laboratories, Detroit, MI, USA) containing solutions of *C. reticulata* EO at 200, 400, 600, 800, or 1000 μL L^−1^ diluted in 0.05% Tween-80. The controls previously described in point 4.3 were also implemented here. Then, treatments and controls were incubated at 25 °C ± 2 °C and 70 rpm on an orbital shaker for 1 week (Labwit, Shanghai, China) for 1 week. Simultaneously, extracellular pH and electrical conductivity measures of the *B. cinerea* cultures were carried out using a portable PCT-407 multiparameter instrument (EZODO, Taipei, Taiwan) [79]. After, the *B. cinerea* cultures were centrifuged at 4000 rpm for 15 min and then, supernatants were discarded. The samples were cleaned with deionized water on a vacuum filtration system and then dried at 50 °C for 48 h to determine the dry weight in an analytical balance (Radwag, Radom, Poland) [79]. Three replicates were conducted for each treatment and controls.

### 4.6. Release of Constituents Absorbing at OD_260nm_ and Soluble Proteins

A solution of 100 μL of spore (10^4^–10^5^ conidia mL^−1^) was inoculated into 900 μL of PDB containing the *C. reticulata* EO at 200, 400, 600, 800, or 1000 μL L^−1^ in 0.05% Tween-80. The controls previously described in point 4.3 were also implemented here. *B. cinerea* cultures were homogenized at 25 °C ± 2 °C and centrifugated at 70 rpm for 2 h and 4 h in an orbital shaker. Then, the fungi cultures were centrifuged at 10,000 rpm for 5 min. Supernatants were used to measure the absorbed constituents at OD_260nm_ and soluble proteins through Bradford methodology [63,80]. Accordingly, 132 μL of Bradford reagent (Sigma-Aldrich, Steinheim, Germany) was added to 68 μL of fungi spore solution. The bovine serum albumin (Sigma-Aldrich, Steinheim, Germany) standard concentrations from 0 to 2 mg mL^−1^ were used to perform a calibration curve. The total protein concentration was measured at 595 nm in a spectrophotometer Epoch (Biotek Instrument, Winooski, VT, USA) integrated with the Gen 5 software (version 2.00.18). Three replicates were implemented for each treatment and control.

### 4.7. Fluorescent Staining to Explore Mitochondrial Activity, Cell Viability, and Esterase Activity of Cultures of B. cinerea

Cultures of *B. cinerea* with 50 mL of PDB containing a solution of *C. reticulata* EO at 1000 μL L^−1^ dissolved in Tween-80 were inoculated with 100 µL of *B. cinerea* spores at 10^4^–10^5^ conidia mL^−1^. The controls described in point 4.3 were used here. Cultures of *B. cinerea* were incubated at 25 °C ± 2 °C in an orbital shaker (200 rpm) for 48 h. The cultures were centrifuged, and then the fungi biomass was washed with phosphate-buffered saline (PBS) 1X (Gibco^®^, Thermo Fisher Scientific, New York, NY, USA). After, 500 µL of samples for each *B. cinerea* culture was extracted for fluorescent staining. Samples were incubated in the following conditions: (1) 0.1 µg mL^−1^ of MitoTracker™ Orange CMTMRos (Invitrogen, Thermo Fisher, Waltham, MA, USA) for 30 min at 30 °C ± 2 °C, (2) 10 µg mL^−1^ of Propidium iodide (Invitrogen, ThermoFisher, Waltham, MA, USA) for 30 min, and (3) 1 µg mL^−1^ of Calcein-AM (Invitrogen^TM^, Thermo Fisher Scientific, USA) [52,81,82]. The intensity of the fluorescence was determined in a multimodal microplate reader model Feyond-a300 (Allsheng Instruments Co., Ltd., Hangzhou, China) using the following excitation and emission wavelengths: 554/575 nm for MitoTracker™ Orange, 535/617 nm for propidium iodide, and 477/520 nm for Calcein-AM. Three replicates were used for each treatment and control.

### 4.8. Succinate Dehydrogenase Activity

Cultures were performed inoculating 100 μL of *B. cinerea* spores (10^4^–10^5^ conidia mL^−1^) in 50 mL of PDB containing the EO of *C. reticulata* at 1000 μL L^−1^ in Tween-80. The controls described in point 4.3 were also used here. The *B. cinerea* cultures were incubated at 25 °C for 72 h and after, centrifuged at 5000 rpm for 10 min. The supernatants were discarded, and spores were collected from the pellet. Next, 300 μL of MTT bromide reagent (ThermoFisher Scientific Inc., Waltham, MA, USA) at 0.5 mg mL^−1^ in PBS was added to the spores. Fungal spore samples were incubated at 25 °C ± 2 °C and centrifuged at 200 rpm for 4 h under dark conditions. Samples were centrifuged at 5000 rpm for 10 min and 300 μL of isopropanol–hydrochloric acid solution (95:5) was added to the pellet [83]. Each solution was analyzed at 560 nm using a spectrophotometer Epoch (Biotek Instrument, Winooski, VT, USA) integrated with the Gen 5 software (version 2.00.18).

### 4.9. Formulation and Characterization of SLN Loaded with the EO of C. reticulata

SLNs were formulated using high-shear homogenization followed by the ultrasonication method. The aqueous phase was Tween-80 at 1.5% w v^−1^ (Difco^®^ Laboratories, Detroit, MI, USA) dissolved in 40 mL of distilled water. The lipid phase was 250 mg of glyceryl tristearate (Sigma-Aldrich^®^, St. Louis, MO, USA) dissolved in 5 mL of *n*-hexane (MerckMilipore^®^, Bedford, MA, USA). Both aqueous and lipid solutions were maintained at 70 °C, and the EO of *C. reticulata* was applied to the lipid phase to obtain a final concentration at 1000 μL L^−1^. The aqueous phase was gently added to the lipid phase, and the resulting solution was homogenized at 10,000 rpm for 5 min by an Ultraturrax OV5 (VELP^®^, Scientifica, Usmate Velate, Italy) and after, sonicated six times for 1 min at 35% amplitude (5 s on/off). The stability of the SLN formulation was confirmed through δ-potential measurements for 3 weeks, with values ranging from −17.7 mV to −25.5 mV. Finally, the formulation of SLN loaded with the EO of *C. reticulata* was stored at 4 °C in a pharmaceutical refrigerator (Refrigerator, 290 L, HYC-290, Haier^®^, Qingdao, China) [84]. The hydrodynamic size, polydispersity index (PDI), and charge surface (ζ-potential) were determined by dynamic dispersion of light (DLS) using a Zetasizer Nano ZS90 (Malvern Instruments, Inc., Malvern, UK) [47]. For morphological characterization, a scanning transmission electron microscopy (STEM) (HITACHI SU3500, Hitachi^®^, Tokyo, Japan) was conducted. To calculate the encapsulation efficiency (*EE*), 30 mL of SLN solution was centrifuged at 9000 rpm for 15 min at 4 °C to separate the lipid fraction from the aqueous solution. Then, 100 mg supernatant was diluted in 2 mL of distilled water (MerckMilipore, Bedford, MA, 275 USA) and analyzed at 285 nm using an Epoch microplate spectrophotometer (BioTek 276 Instruments, Winooski, VT, USA). A calibration curve was prepared using 50, 100, 200, 250, 277, 500, and 750 μL L^−1^ of *C. reticulata* EO dissolved in *n*-hexane to obtain the regression equation. *EE* was determined using Equation (1), where *Wa* is the concentration of EO applied to formulate SLN and *Ws* is the concentration quantified in the supernatant [84].

(1) $$EE\%=\left(\frac{Wa-Ws}{wa}\right)\times 100$$

### 4.10. Antifungal Activity of SLN Loaded with the EO of C. reticulata

Fungi mycelia disks of 5 mm in diameter were extracted from the edges of a *B. cinerea* colony culture growth during 7 days on PDA (Difco^®^ Laboratories, Detroit, MI, USA). The *B. cinerea* mycelia disk was placed on the surface of the PDA medium supplemented with the *C. reticulata* EO-loaded SLN at 1000 μL L^−1^. Untreated plates (without EO-loaded SLN) were considered the first control, and plates with SLN formulation (without EO) were the second control. Then, the plates were sealed with Parafilm (Heathrow Scientific™, Vernon Hills, IL, USA) and incubated at 25 °C ± 2 °C for 4 days.

### 4.11. Statistical Analysis

All bioassays were performed with three replicates for each treatment and control. The data were subjected to an analysis of variance (ANOVA) and mean separations were performed using a Tukey test (*p* ≤ 0.05), using Statistix v10 software.

## Figures and Tables

**Table 1:** Chemical composition of the *Citrus* essential oils.

RT	RI	Compound	*C. reticulata* (%)	*C. limon* (%)	*C. paradisi* (%)	*C. sinensis* (%)	Identification
8.65	929	*α*-Pinene	2.54	2.58	0.57	0.42	RI, MS, Std-I
9.05	943	Camphene	-	0.03	-	-	RI, MS
9.83	969	*β*-Pinene	2.23	**21.89**	0.12	0.14	RI, MS, Std-I
10.79	998	*β*-Myrcene	0.61	0.61	0.35	0.18	RI, MS
11.33	1017	*o*-Cymene	1.08	0.17	-	-	RI, MS
11.51	1024	**Limonene**	**66.61**	**59.65**	**98.51**	**95.23**	RI, MS, Std-I
12.37	1054	*γ*-Terpinene	**26.29**	**12.85**	-	-	RI, MS, Std-I
15.05	1144	Limonene epoxide	-	-		**3.08**	RI, MS
19.32	1294	Carvone	-	-	-	0.83	RI, MS
21.60	1382	Copaene	0.10	-	0.09	0.03	RI, MS
22.63	1422	Caryophyllene	0.06	0.66	0.21		RI, MS
22.92	1434	*β*-copaene	0.03		0.06	0.01	RI, MS
23.09	1441	*α*-Bergamotene	-	0.68	-	-	RI, MS
23.49	1457	Humulene	0.02	0.03	0.02	-	RI, MS
24.49	1497	Valencene	-	0.05	-	-	RI, MS
26.49	1582	Caryophyllene oxide	0.07	0.09	0.02	-	RI, MS
Identified compounds			99.64	99.29	99.95	99.92	
**Total Monoterpenes**			99.36	97.78	99.55	99.88	
Monoterpene hydrocarbons			99.36	97.78	99.55	95.97	
Oxygenated monoterpenes			-	-	-	3.91	
**Total Sesquiterpenes**			0.28	1.51	0.40	0.04	
Sesquiterpene hydrocarbons			0.21	1.42	0.38	0.04	
Oxygenated sesquiterpenes			0.07	0.09	0.02	-	

RT: retention time (min), RI: calculated Kovats retention index, %: considering detected compounds, Std-I: standard injection. The bold numbers and names indicate majority compounds.

## Data Availability

Data are contained within the article.

## Abbreviations

The following abbreviations are used in this manuscript:

EO	Essential oil
SLN	Solid lipid nanoparticle
GC/MS	Gas–liquid chromatography coupled to mass spectrometry
OD	Optical density
PDI	Polydispersity index
STEM	Scanning transmission electron microscopy
GRAS	Generally recognized as safe
TCA	Tricarboxylic acid
ATP	Adenosine triphosphate
MTT	3- (4,5-Dimethylthiazol-2-yl) 2,5-diphenyltetrazolium
FDA	Food and Drug Administration
PDA	Potato dextrose agar
PDB	Potato dextrose broth
PBS	Phosphate-buffered saline
